# Early- Versus Newer-Generation Transcatheter Mitral Valve Edge-to-Edge Repair Systems: Insights From the OCEAN-Mitral Registry

**DOI:** 10.1016/j.jacasi.2025.05.013

**Published:** 2025-07-15

**Authors:** Taishi Okuno, Masaki Izumo, Shingo Kuwata, Yoshihiro J. Akashi, Masanori Yamamoto, Shunsuke Kubo, Mike Saji, Yuki Izumi, Yusuke Enta, Shinichi Shirai, Shingo Mizuno, Yusuke Watanabe, Makoto Amaki, Kazuhisa Kodama, Junichi Yamaguchi, Toru Naganuma, Hiroki Bota, Yohei Ohno, Masahiko Asami, Daisuke Hachinohe, Masahiro Yamawaki, Hiroshi Ueno, Kazuki Mizutani, Toshiaki Otsuka, Kentaro Hayashida, OCEAN-Mitral Investigators

**Affiliations:** a Department of Cardiology, St Marianna University School of Medicine, Kawasaki, Japan; b Department of Cardiology, Toyohashi Heart Center, Toyohashi, Japan; c Department of Cardiology, Gifu Heart Center, Gifu, Japan; d Department of Cardiology, Nagoya Heart Center, Nagoya, Japan; e Department of Cardiology, Kurashiki Central Hospital, Kurashiki, Japan; f Department of Cardiology, Sakakibara Heart Institute, Tokyo, Japan; g Division of Cardiovascular Medicine, Department of Internal Medicine, Toho University Faculty of Medicine, Tokyo, Japan; h Department of Cardiology, Sendai Kosei Hospital, Sendai, Japan; i Division of Cardiology, Kokura Memorial Hospital, Kitakyushu, Japan; j Department of Cardiology, Shonan Kamakura General Hospital, Kanagawa, Japan; k Department of Cardiology, Teikyo University School of Medicine, Tokyo, Japan; l Department of Cardiology, National Cerebral and Cardiovascular Center, Suita, Japan; m Division of Cardiology, Saiseikai Kumamoto Hospital Cardiovascular Center, Kumamoto, Japan; n Department of Cardiology Tokyo Woman’s Medical University, Tokyo, Japan; o Department of Cardiology, New Tokyo Hospital, Chiba, Japan; p Department of Cardiology, Sapporo Higashi Tokushukai Hospital, Sapporo, Japan; q Department of Cardiology, Tokai University School of Medicine, Isehara, Japan; r Division of Cardiology, Mitsui Memorial Hospital, Tokyo, Japan; s Department of Cardiology, Sapporo Heart Center, Sapporo Cardio Vascular Clinic, Sapporo, Japan; t Department of Cardiology, Saiseikai Yokohama City Eastern Hospital, Kanagawa, Japan; u Second Department of Internal Medicine, Toyama University Hospital, Toyama, Japan; v Division of Cardiology, Department of Medicine, Kindai University Faculty of Medicine, Osaka, Japan; w Department of Hygiene and Public Health, Nippon Medical School, Tokyo, Japan; x Department of Cardiology, Keio University School of Medicine, Tokyo, Japan

**Keywords:** degenerative mitral regurgitation, functional mitral regurgitation, MitraClip, mitral valve repair, transcatheter edge-to-edge repair

## Abstract

**Background:**

Comparative data on early- (G2) vs newer-generation (G4) MitraClip transcatheter edge-to-edge repair (TEER) systems remain limited.

**Objectives:**

The authors compared procedural and clinical outcomes of both devices in patients with degenerative mitral regurgitation (DMR) or functional mitral regurgitation (FMR).

**Methods:**

Using the OCEAN (Optimized Catheter Valvular Intervention)-Mitral registry, 3,738 patients undergoing TEER with either G2 (n = 1,481) or G4 (n = 2,257) MitraClips were analyzed. Outcomes included procedural metrics, echocardiographic parameters, and clinical events.

**Results:**

Despite being older (*P* = 0.010) and higher-risk (EuroSCORE [European System for Cardiac Operative Risk Evaluation] II: *P* = 0.002), the newer-generation group achieved comparable procedural success (residual MR ≤2+) with shorter procedure time and fewer clips, resulting in significantly lower transmitral mean pressure gradients (TMPG) (DMR: median: 3.0 [Q1-Q3: 2.0-4.0] mm Hg vs 2.7 [Q1-Q3: 2.0-3.8] mm Hg; *P* = 0.009; FMR: median 3.0 [Q1-Q3: 2.0-4.0] mm Hg vs 2.5 [Q1-Q3: 1.9-3.5] mm Hg; *P* < 0.001) in both DMR and FMR cohorts. Rates of mechanical complications, such as single leaflet device attachment and leaflet tear, were rare across both device generations but were further reduced in the newer-generation device. The newer-generation device was associated with reduced heart failure (HF) rehospitalization in DMR (adjusted HR: 0.51; 95% CI: 0.33-0.77; *P* = 0.001) and FMR (adjusted HR: 0.76; 95% CI: 0.61-0.93; *P* = 0.009), with a greater risk reduction in DMR (*P*_interaction_ < 0.001). A causal mediation analysis revealed that postprocedural TMPG modestly mediated the association between device generation and HF rehospitalization risk (proportion mediated = 1.8% [Q1-Q3: 0.3%-4.0%]; *P* = 0.016).

**Conclusions:**

The newer-generation TEER system offers a safer and more efficient procedure, with shorter procedural time, fewer mechanical complications, fewer clips, and a lower postprocedural TMPG, contributing to reduced HF rehospitalization risk, particularly in DMR.

Mitral regurgitation (MR) is the most prevalent valvular heart disease, affecting over 20 million people globally, with a pronounced impact on aging populations. Transcatheter mitral valve edge-to-edge repair (M-TEER) has established itself as a viable therapeutic option for symptomatic patients with severe degenerative mitral regurgitation (DMR) who are at high or prohibitive surgical risk,^1^ as well as selected patients with symptomatic heart failure (HF) and functional mitral regurgitation (FMR) receiving optimal medical therapy.^2^, ^3^, ^4^ Over the years, growing experience and advancements in device technology have continued to improve the procedural outcomes of M-TEER. Recently, the MitraClip G4 system has been introduced, offering remarkable advancements such as 4 different clips sizes and independent grasping capabilities, and is currently in use worldwide. The MitraClip EXPAND G4 study (NCT04177394), a postmarket, multicenter, single-arm, prospective study, reported promising results demonstrating high and durable reductions in MR, low all-cause mortality, and improved functional status.^5^^,^^6^ Because real-world clinical data comparing early-generation (G2) vs the newer-generation (G4) M-TEER systems remain limited, this study aims to compare patient profiles, procedures, and clinical outcomes between those treated with these 2 generations of systems in a large-scale, nation-wide, multicenter cohort of patients undergoing M-TEER.

## Methods

### Study cohort

The OCEAN (Optimized Catheter Valvular Intervention)-Mitral registry is an ongoing investigator-initiated, prospective, multicenter cohort study designed to include patients with significant MR undergoing M-TEER at collaborating centers in Japan.^7^^,^^8^ Continuous patient enrollment and adherence to the study protocol, including the provision of patient data and clinical outcomes during follow-up, are mandatory for all centers involved. The Ethics Committees at each center approved the study protocol, and all participants gave written informed consent. The study complies with the Declaration of Helsinki and is registered with the University Hospital Medical Information Network Clinical Trial Registry (UMIN000023653). This analysis included all patients who underwent M-TEER with either the MitraClip G2 or G4 between April 2018 and June 2023.

### Procedure

All procedures were conducted under the guidance of the local Heart Team in accordance with established guidelines.^9^ Transthoracic echocardiography was performed at baseline and repeated postprocedure, before discharge, and at follow-up visits (at 1 month and 1 year) by experienced echocardiographers. The procedure was performed under general anesthesia with 2- and 3-dimensional transesophageal echocardiographic and fluoroscopic guidance. The MitraClip G4 system, introduced in Japan in September 2020, offers 4 clip sizes and allows independent leaflet grasping. Clip size was chosen by the local Heart Team based on anatomical assessments using transesophageal echocardiography during the procedure.

### Data collection and definitions

Baseline, procedural, and follow-up data were prospectively collected and entered into a centralized web-based database at each participating center. Follow-up assessments were scheduled at 1 month, 1 year, and annually thereafter. Clinical follow-up data were obtained through medical records, communication with referring physicians, and/or telephone interviews. A Data Committee conducted regular audits of the database to ensure completeness and accuracy, and centers were required to respond to any data queries.

MR severity was graded on a scale of 0-4+ in accordance with the American Society of Echocardiography guidelines.^10^ For patients with DMR, flail gap and flail width were evaluated, whereas coaptation length and coaptation depth were assessed for those with FMR, following a standardized protocol. In the case of FMR, atrial FMR was defined as FMR occurring in patients with atrial fibrillation, an enlarged left atrium, and preserved left ventricular function and size.^11^ Acute procedural success was defined as successful device implantation resulting in MR severity of 2+ or less at discharge. Clinical outcomes of interest were the composite endpoint of all-cause mortality or HF rehospitalization, and its components.

### Statistical analysis

Categorical data are represented as frequencies and percentages, with differences between groups evaluated using the chi-square test or Fisher exact test. All continuous variables were assessed for normality using the Shapiro-Wilk test, and because they deviated significantly from a normal distribution (*P* < 0.05), these variables are expressed as median values with Q1-Q3 ranges and compared between groups using Mann-Whitney *U* test. Clinical events are presented in a time-to-event manner utilizing the Kaplan-Meier method and compared using the log-rank test, whereas Cox proportional hazards models were employed to calculate HRs and 95% CIs. Because some Kaplan-Meier curves exhibited overlap, we performed a post hoc check of the proportional hazards assumption using Schoenfeld residuals for all endpoints, and found no significant violations (*P* ≥ 0.05). Patients were censored to the time of last known follow-up or death. Given the fundamental difference between DMR and FMR, a multivariable adjustment was conducted using separate independent models for the DMR and FMR cohorts, respectively. Baseline variables included in each model are listed in Supplemental Table 1.

To ensure the robustness of the findings, sensitivity analyses were performed. First, cases from the first year of M-TEER adoption in Japan (2018) were excluded to account for potential learning curve effects. Second, propensity score matching was conducted to further validate the results. The propensity score was estimated using a multivariable logistic regression model, incorporating the same variables used in the Cox multivariable adjustment models (Supplemental Table 1). Matching was performed using a 1:1 greedy nearest-neighbor algorithm, with a caliper width of 0.2.

Interaction analysis using Cox proportional hazards models was conducted to assess whether interactions between variables modified the risk of clinical outcomes. A causal mediation analysis was conducted to investigate whether the observed relationship between device generation and clinical outcomes was mediated through procedural outcomes. The analysis estimated the average causal mediation effect and average direct effect, with nonparametric bootstrapping (1,000 iterations) employed to derive confidence intervals.

Throughout this study, *P* < 0.05 was considered statistically significant. All statistical analyses were conducted using EZR software (Saitama Medical Center, Jichi Medical University) with R coding executed to supplement advanced statistical procedures (R version 4.2.1, The R Foundation for Statistical Computing).

## Results

### Study population and baseline characteristics

During the study period, 3,738 patients underwent M-TEER using the MitraClip system at 21 collaborating centers. Of these, 1,481 were treated with the G2 system, and 2,257 with the G4 system. Baseline characteristics are detailed in Table 1. Overall, patients in the newer-generation group tended to be older (median: 80 [Q1-Q3: 73-85] years vs 81 [Q1-Q3: 74-86] years; *P* = 0.010) and had higher surgical risk scores (median: EuroSCORE [European System for Cardiac Operative Risk Evaluation] II: 4.86 [Q1-Q3: 3.06-8.21] vs 5.14 [Q1-Q3: 3.33-9.19]; *P* = 0.002) compared to the early-generation group. There were differences in comorbidities, with lower rates of hypertension (67.5% vs 63.6%; *P* = 0.016), diabetes (28.2% vs 25.1%; *P* = 0.037), dyslipidemia (50.8% vs 46.8%; *P* = 0.016), atrial fibrillation (63.7% vs 60.3%; *P* = 0.036), coronary artery disease (36.9% vs 33.7%; *P* = 0.046), and chronic obstructive pulmonary disease (10.3% vs 7.4%; *P* = 0.002) in the newer-generation group.

**Table 1:** Baseline Characteristics All Cohorts (N = 3,738)

	Early-Generation (n = 1,481)	Newer-Generation (n = 2,257)	*P* Value
Age, y	80 (73-85)	81 (74-86)	0.010
Male	833 (56.2)	1,222 (54.1)	0.214
Body mass index, kg/m^2^	21.0 (18.8-23.3)	21.0 (18.7-23.2)	0.838
Body surface area, m^2^	1.50 (1.37-1.64)	1.50 (1.36-1.65)	0.928
EuroSCORE II	4.86 (3.06-8.21)	5.14 (3.33-9.19)	0.002
Clinical frailty scale	4 (3-5)	4 (3-4)	0.141
NYHA functional class III or IV	957 (64.6)	1,405 (62.3)	0.145
Concomitant diseases			
Hypertension	999 (67.5)	1,435 (63.6)	0.016
Diabetes mellitus	417 (28.2)	566 (25.1)	0.037
Dyslipidemia	753 (50.8)	1,056 (46.8)	0.016
Chronic kidney disease (eGFR <60)	1,279 (86.4)	1,959 (86.8)	0.731
Atrial fibrillation	943 (63.7)	1,360 (60.3)	0.036
Coronary artery disease	546 (36.9)	760 (33.7)	0.046
COPD	153 (10.3)	168 (7.4)	0.002
Etiology of mitral regurgitation			0.715
Degenerative mitral regurgitation	447 (30.2)	669 (29.6)	
Functional mitral regurgitation	1,034 (69.8)	1,589 (70.4)	

Values are median (Q1-Q3) or n (%).

COPD = chronic obstructive pulmonary disease; eGFR = estimated glomerular filtration rate; EuroSCORE = European System for Cardiac Operative Risk Evaluation.

Among the overall cohort, 1,115 patients (29.8%) had DMR and 2,623 (70.2%) had FMR. Supplemental Table 2 provides additional baseline comparisons within the DMR and FMR subgroups. Data on optimal medical therapy in the FMR cohort, including guideline-directed medical therapy and cardiac resynchronization therapy, are shown in Supplemental Table 3. The use of renin-angiotensin system inhibitors was similar between groups (65.0% vs 65.5%; *P* = 0.833). However, an angiotensin receptor-neprilysin inhibitor was predominantly used in the newer-generation group (0.1% vs 20.7%; *P* < 0.001). Likewise, sodium-glucose cotransporter-2 inhibitors were used more frequently in the newer-generation group (10.1% vs 35.4%; *P* < 0.001). In contrast, the newer-generation group had lower usage rates of beta-blockers (83.3% vs 79.3%; *P* = 0.011) and loop diuretics (86.5% vs 78.2%; *P* < 0.001). Cardiac-resynchronization therapy devices were less frequently implanted in the newer-generation group (15.0% vs 12.3%; *P* = 0.046).

### Echocardiographic characteristics

Baseline echocardiographic characteristics of the DMR and FMR cohorts are shown in Table 2. Among DMR patients, anatomical complexity—such as the presence of bileaflet prolapse or noncentral jet, leaflet length, flail gap, and flail width—was similar across device generations. However, the newer-generation group had a smaller mitral valve area (median: 5.2 [Q1-Q3: 4.3-6.2] cm^2^ vs 4.8 [Q1-Q3: 4.1-5.9] cm^2^; *P* = 0.001) and a higher transmitral mean pressure gradient (TMPG) (median: 2.0 [Q1-Q3: 1.0-2.7] mm Hg vs 2.0 [Q1-Q3: 1.3-3.0] mm Hg; *P* = 0.012). In the FMR cohort, although anatomical complexity regarding leaflet length, coaptation length, and coaptation depth was similar between device generations, the newer-generation group exhibited smaller left ventricular dimensions (median left ventricular end-diastolic volume: 155.0 [Q1-Q3: 111.0-206.0] mL vs 145.8 [Q1-Q3: 101.9-201.0] mL; *P* = 0.010; median left ventricular end-systolic volume: 101.0 [Q1-Q3: 63.0-143.0] mL vs 95.0 [Q1-Q3: 53.0-144.0] mL; *P* = 0.039) and smaller mitral valve area (median: 5.1 [Q1-Q3: 4.2-6.2] cm^2^ vs 5.0 [Q1-Q3: 4.0-6.1] cm^2^; *P* = 0.021).

**Table 2:** Echocardiographic Characteristics in the DMR and FMR Cohorts

	Early-Generation	Newer-Generation	*P* Value
DMR cohort (N = 1,115), n	447	668	
LVEF, %	62.9 (55.4-67.2)	62.3 (55.3-67.2)	0.913
MR grade ≥3+	420 (94.0)	636 (95.2)	0.413
Bileaflet prolapse	93 (20.8)	130 (19.5)	0.593
Noncentral jet	176 (39.4)	291 (43.6)	0.173
Posterior leaflet length, mm	12.4 (10.1-15.0)	12.0 (10.0-14.3)	0.082
Flail gap, mm	5.8 (3.8-7.6)	5.2 (3.7-7.3)	0.058
Flail gap ≥10 mm	31 (9.2)	37 (8.6)	0.800
Flail width, mm	11.9 (9.2-14.8)	12.0 (9.1-14.8)	0.685
Flail width ≥15 mm	80 (24.4)	95 (24.3)	>0.999
Mitral valve area, cm^2^	5.2 (4.3-6.2)	4.8 (4.1-5.9)	0.001
TMPG, mm Hg	2.0 (1.0-2.7)	2.0 (1.3-3.0)	0.012
FMR cohort (N = 2,623), n	1,034	1,589	
LVEF, %	35.5 (29.3-47.6)	35.3 (26.9-48.9)	0.397
LVEDV, mL	155.0 (111.0-206.0)	145.8 (101.9-201.0)	0.010
LVESV, mL	101.0 (63.0-143.0)	95.0 (53.0-144.0)	0.039
LA volume index, mL/m2	67.7 (59.3-105.2)	71.8 (56.0-97.2)	<0.001
MR grade ≥3+	897 (86.8)	1293 (81.4)	<0.001
Atrial FMR	188 (18.2)	335 (21.1)	0.072
Posterior leaflet length, mm	11.5 (9.6-13.5)	11.4 (9.6-13.7)	0.584
Coaptation length, mm	2.8 (2.0-3.6)	2.6 (2.0-3.5)	0.079
Coaptation length >2 mm	610 (82.4)	689 (79.1)	0.100
Coaptation depth, mm	7.7 (5.3-10.0)	7.5 (5.1-9.9)	0.318
Coaptation depth <11 mm	722 (82.8)	949 (82.2)	0.768
Mitral valve area, cm^2^	5.1 (4.2-6.2)	5.0 (4.0-6.1)	0.021
TMPG, mm Hg	1.6 (1.0-2.0)	1.5 (1.0-2.1)	0.114

Values are median (Q1-Q3) or n (%), unless otherwise indicated.

DMR = degenerative mitral regurgitation; FMR = functional mitral regurgitation; LA = left atrium; LVEDV = left ventricular end-diastolic volume; LVEF = left ventricular ejection fraction; LVESV = left ventricular end-systolic volume; MR = mitral regurgitation; TMPG = transmitral mean pressure gradient.

### Procedural characteristics and outcomes

**Figure 1:**
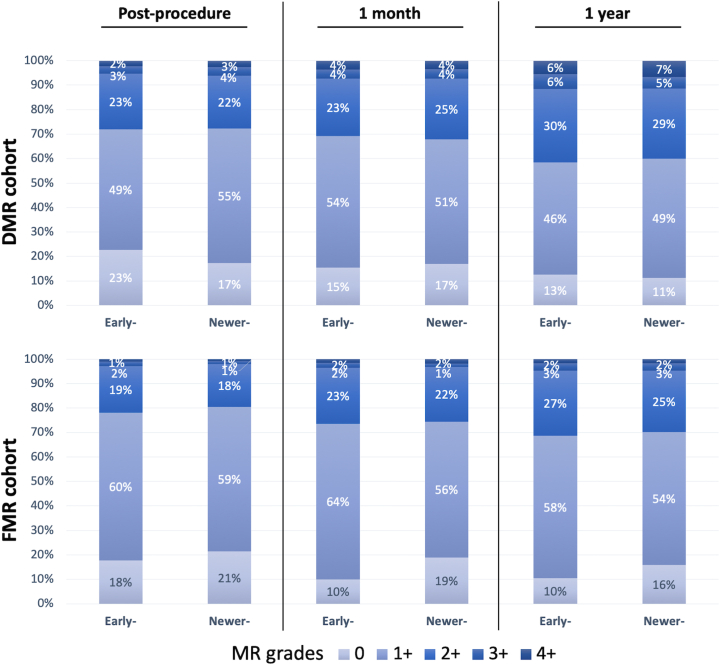
MR Grades During Follow-Up Bar graphs comparing mitral regurgitation (MR) severity between early- and newer-generation transcatheter mitral valve edge-to-edge repair systems at discharge, 1 month, and 1 year. The progressively darker shades of blue correlate with increasing grades of MR, ranging from 0 to 4+. DMR = degenerative mitral regurgitation; FMR = functional mitral regurgitation.

Details of procedural characteristics and outcomes are listed in Table 3. The use of the newer-generation device was associated with significantly shorter procedural time (median DMR: 93 [Q1-Q3: 67-128] min vs 80 [Q1-Q3: 60-110] min; *P* < 0.001 median: FMR: 91 [Q1-Q3: 65-125] min vs 73 [Q1-Q3: 55-97] min; *P* < 0.001) and device time (median DMR: 66 [Q1-Q3: 43-97] min vs 53 [Q1-Q3: 39-80] min; *P* < 0.001; median: FMR: 64 [Q1-Q3: 43-90] min vs 45 [Q1-Q3: 34-63] min; *P* < 0.001), as well as a reduced number of clips used in both the DMR and FMR cohorts. Acute procedural success was achieved in approximately 93% of the DMR cohort and 97% of the FMR cohort, with no differences observed between device generations. Mechanical complications, including single leaflet device attachment, leaflet tear, and clip embolization, were rare across both groups, but were even lower in the newer-generation group for both the DMR and FMR cohorts (Table 3). At discharge, MR severity was similar between early-generation and newer-generation systems (Figure 1). However, TMPG was significantly lower in the newer-generation group compared to the early-generation group in both the DMR cohort (median: 3.0 [Q1-Q3: 2.0-4.0] mmHg vs 2.7 [Q1-Q3: 2.0-3.8] mm Hg; *P* = 0.011) and the FMR cohort (median: 3.0 [Q1-Q3: 2.0-4.0] mm Hg vs 2.5 [Q1-Q3: 1.9-3.5] mm Hg; *P* < 0.001).

**Table 3:** Procedural Characteristics and Outcomes in the DMR and FMR Cohorts

	Early-Generation	Newer-Generation	*P* Value
DMR cohort (N = 1,115), n	447	668	
Procedural time, min	93 (67-128)	80 (60-110)	<0.001
Device time, min	66 (43-97)	53 (39-80)	<0.001
No. of clips			<0.001
1	230 (51.5)	498 (74.6)	
2	207 (46.3)	163 (24.4)	
3	10 (2.2)	7 (1.0)	
No. of clips	1 (1-2)	1 (1-2)	<0.001
Extended arm clips (XT/XTW)	NA	330 (49.4)	
Wide clips (NTW/XTW)	NA	476 (71.2)	
Procedural outcome			
Acute procedural success (MR ≤2+)	416 (93.1)	619 (92.7)	0.906
SLDA	12 (2.7)	9 (1.3)	0.119
Leaflet tear	8 (1.8)	2 (0.3)	0.018
Clip embolization	3 (0.7)	2 (0.3)	0.395
Echocardiographic outcome at discharge			
MR ≥3+	24 (5.4)	40 (6.0)	0.696
TMPG, mm Hg^a^	3.0 (2.0-4.0)	2.7 (2.0-3.8)	0.009
TMPG ≥5 mm Hg^a^	55 (13.5)	59 (9.7)	0.068
FMR cohort (N = 2,623), n	1,034	1,589	
Procedural time, min	91 (65-125)	73 (55-97)	<0.001
Device time, min	64 (43-90)	45 (34-63)	<0.001
No. of clips			<0.001
1	593 (57.4)	1,387 (87.3)	
2	423 (40.9)	192 (12.1)	
3	18 (1.7)	35 (2.2)	
No. of clips	1 (1-2)	1 (1-1)	<0.001
Extended arm clips (XT/XTW)	NA	560 (35.2)	
Wide clips (NTW/XTW)	NA	1,239 (78.0)	
Procedural outcome			
Acute procedural success (MR ≤2+)	996 (96.3)	1,548 (97.4)	0.128
SLDA	16 (1.5)	11 (0.7)	0.046
Leaflet tear	20 (1.9)	14 (0.9)	0.022
Clip embolization	0 (0)	0 (0)	>0.999
Echocardiographic outcome at discharge			
MR ≥3+	30 (2.9)	32 (2.0)	0.188
TMPG, mm Hg^a^	3.0 (2.0-4.0)	2.5 (1.9-3.5)	<0.001
TMPG ≥5 mm Hg^a^	108 (11.5)	130 (9.2)	0.070

Values are n (%) or median (Q1-Q3), unless otherwise indicated.

NA = not available; SLDA = single leaflet device attachment; other abbreviations as in Table 2.

a Outliers were identified and excluded using the Smirnov-Grubbs test.

MR severity at follow-up is presented in Figure 1. There were no significant differences in the rate of residual MR (>2+) between device generations at 1 month and 1 year, in either the DMR or FMR cohort.

### Clinical outcomes

**Figure 2:**
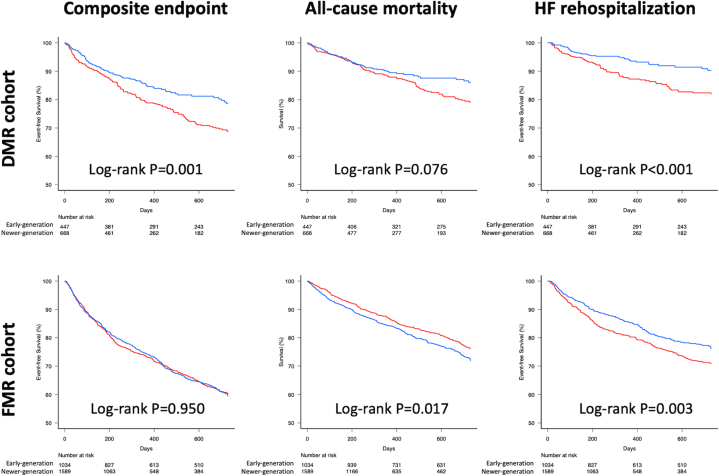
Clinical Outcomes of Early- vs Newer-Generation M-TEER System Kaplan-Meier curves illustrating 2-year event-free survival rates in patients treated with early- (red) and newer-generation (blue) transcatheter mitral valve edge-to-edge repair (M-TEER) systems. The composite endpoint includes all-cause mortality and heart failure (HF) rehospitalization. Abbreviations as in Figure 1.

The results of clinical outcomes are presented in Table 4. The average follow-up time was 830 ± 494 days for the early-generation group and 419 ± 299 days for the newer-generation group. In the DMR cohort, the newer-generation device was associated with a significantly lower risk of the composite endpoint (all-cause mortality or HF rehospitalization) compared to the early-generation device (adjusted HR: 0.68; 95% CI: 0.50-0.88; *P* = 0.005). However, in the FMR cohort, the risk was similar between device generations (adjusted HR: 0.99’ 95% CI: 0.84-1.16; *P* = 0.869). No significant difference in mortality risk was observed in the DMR cohort (adjusted HR: 0.78; 95% CI: 0.55-1.10; *P* = 0.154) or FMR cohort (adjusted HR: 1.22; 95% CI: 1.00-1.49; *P* = 0.055). Notably, the incidence of HF rehospitalization was lower with the newer-generation system in both the DMR (adjusted HR: 0.51; 95% CI: 0.33-0.77; *P* = 0.001) and FMR cohorts (adjusted HR: 0.76; 95% CI: 0.61-0.93; *P* = 0.009) (Figure 2). Furthermore, significant interactions were observed across all evaluated clinical endpoints (Supplemental Figure 1), indicating that the protective effect of the newer-generation system was significantly greater in the DMR cohort than in the FMR cohort.

**Table 4:** Clinical Outcomes at 2-year Follow-Up

	Early-Generation	Newer-Generation	HR (95% CI)	*P* Value	Adjusted HR^a^	*P* Value^a^
DMR cohort (N = 1,115), n	447	668				
Composite endpoint of mortality and HF rehospitalization	182 (31.3)	103 (21.3)	0.66 (0.51-0.85)	0.001	0.67 (0.50-0.88)	0.005
All-cause mortality	130 (20.8)	71 (14.0)	0.76 (0.55-1.03)	0.077	0.78 (0.55-1.10)	0.154
Cardiovascular mortality	73 (11.3)	39 (7.8)	0.78 (0.51-1.20)	0.259	0.80 (0.49-1.28)	0.349
HF rehospitalization	97 (18.0)	41 (9.7)	0.48 (0.33-0.70)	<0.001	0.51 (0.33-0.77)	0.001
FMR cohort (N = 2,623), n	1,034	1,589				
Composite endpoint of mortality and HF rehospitalization	500 (39.6)	464 (40.5)	1.00 (0.88-1.15)	0.951	0.99 (0.84-1.16)	0.869
All-cause mortality	356 (23.7)	313 (28.1)	1.22 (1.04-1.44)	0.017	1.22 (1.00-1.49)	0.055
Cardiovascular mortality	214 (15.3)	188 (18.3)	1.22 (0.99-1.51)	0.064	1.06 (0.82-1.38)	0.645
HF rehospitalization	330 (29.1)	243 (23.8)	0.77 (0.65-0.91)	0.003	0.76 (0.61-0.93)	0.009

Values are n (%) unless otherwise indicated. Depicted are the number of events (counting only the first event per patient), with Kaplan-Meier cumulative incidences presented as percentages in parentheses at 2 years (730 days). Patients were censored at the time of last known follow-up or death.

HF = heart failure; other abbreviations as in Table 2.

a Adjusted HRs and *P* values were calculated using multivariable analyses with separate models for the DMR and FMR cohorts (see Supplemental Table 1).

Results of sensitivity analyses, which excluded cases from the first year of M-TEER adoption in Japan (2018), as well as those in the propensity score-matched cohorts, yielded consistent findings (Supplemental Tables 4 and 5).

**Figure 3:**
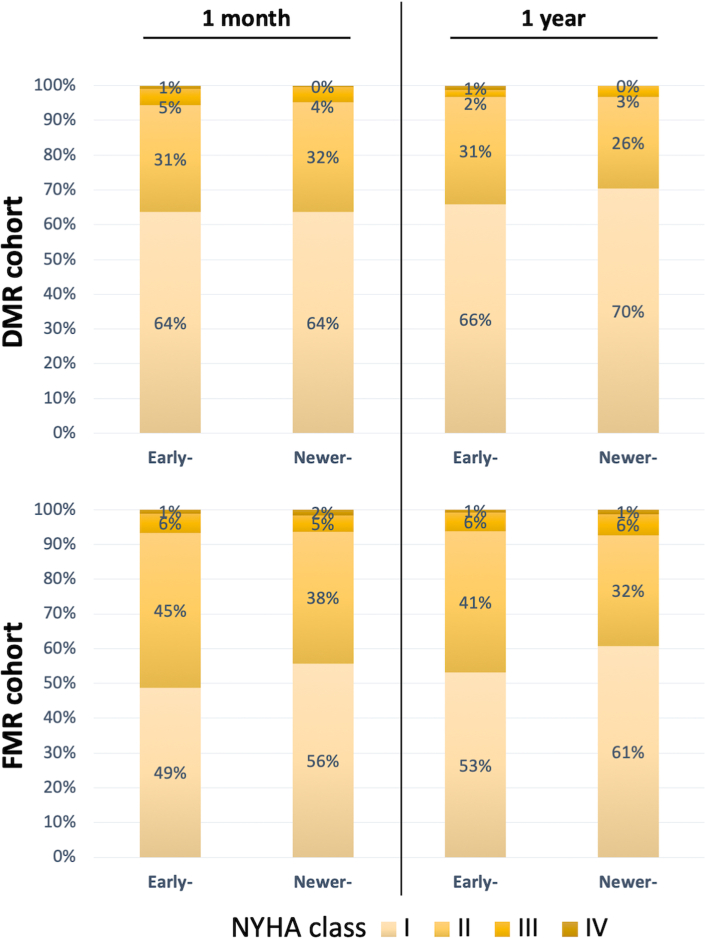
NYHA Functional Class at Follow-Up Bar graphs comparing NYHA functional class at 1 month and 1 year. The progressively darker shades of yellow correlate with increasing grades of NYHA functional class from I to IV. Abbreviations as in Figure 1.

In the DMR cohort, residual heart failure symptoms (NYHA functional class ≥ 3) were documented in approximately 5%-6% of patients at 1 month and 3% at 1 year, whereas in the FMR cohort, these symptoms were present in 6%-7% of patients at both 1 month and 1 year, with no significant differences between device generations (Figure 3).

### Relationship between procedural outcomes and HF rehospitalization

TMPG at discharge was significantly associated with the risk of HF rehospitalization (HR: 1.10; 95% CI: 1.04-1.15; *P* < 0.001). However, no significant interaction effect was found between device generation and TMPG at discharge regarding the risk of HF rehospitalization (*P*_interaction_ = 0.199). Higher MR grades at discharge were also associated with an increased risk of HF rehospitalization (HR: 1.21; 95% CI: 1.10-1.33; *P* < 0.001). Nonetheless, the interaction between device generation and MR severity regarding the risk of HF rehospitalization did not reach statistical significance (*P*_interaction_ = 0.191).

Causal mediation analysis revealed that the TMPG at discharge significantly mediated the association between device generation and HF rehospitalization, though the mediation effect was modest, accounting for only 1.8% of the total effect (*P* = 0.016). In contrast, MR severity at discharge did not significantly mediate this association (*P* = 0.220) (Supplemental Figure 2).

Supplemental Figure 3 presents subgroup analyses on the impact of discharge TMPG and MR severity on HF rehospitalization stratified by MR etiology. The analyses revealed no significant interaction between TMPG and MR etiology on HF rehospitalization risk (*P*_interaction_ = 0.750), whereas a significant interaction was observed for MR severity (*P*_interaction_ < 0.001), with a greater impact on the DMR cohort.

## Discussion

The salient findings from this large-scale, multicenter registry can be summarized as follows (Central Illustration):

• Although baseline anatomical characteristics of the mitral valve were generally comparable, patients with a smaller mitral valve area were more likely to be treated with the newer-generation M-TEER system.
• The newer-generation device was associated with shorter procedural and device times, a reduced number of clips, and maintained a similarly high rate of acute procedural success (MR ≤2+) compared to the early-generation device. Importantly, postprocedural TMPG was significantly lower in the newer-generation group.
• Mechanical complications, including single leaflet device attachment, leaflet tear, and clip embolization, were infrequent in both device groups, with further reduction in the newer-generation device.
• Use of the newer-generation M-TEER system was significantly associated with a lower risk of HF rehospitalization during follow-up, with a more pronounced protective effect in the DMR cohort compared to the FMR cohort.
• The reduced risk of HF rehospitalization in the newer-generation device was partially explained by the lower postprocedural TMPG.

**Central Illustration:**
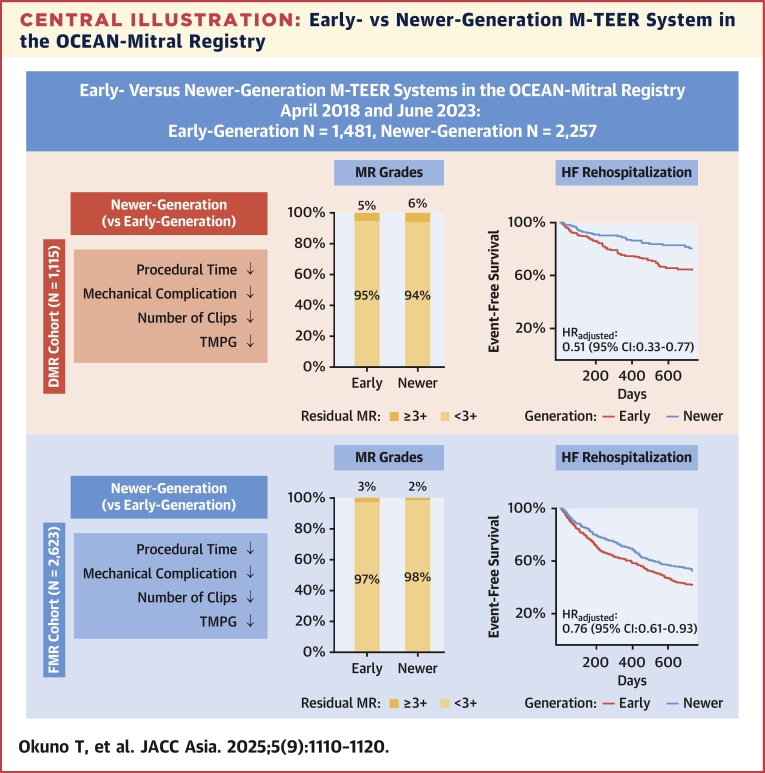
Early- vs Newer-Generation M-TEER System in the OCEAN-Mitral Registry Illustration showing that the newer-generation transcatheter mitral valve edge-to-edge repair (M-TEER) system reduced procedural time and the number of clips required per procedure, achieving a lower transmitral mean pressure gradient (TMPG) and comparable mitral regurgitation (MR) reduction to the early-generation system. Residual MR (≥3+) was observed in 5.4% of the early-generation group and in 6.0% of the newer-generation group in the degenerative mitral regurgitation (DMR) cohort, whereas in 2.9% of the early-generation group and in 2.0% of the newer-generation group in the functional mitral regurgitation (FMR) cohort. The newer-generation device demonstrated a reduced risk of heart failure (HF) rehospitalization, with a more pronounced protective effect in patients with DMR than those with FMR. OCEAN-Mitral Registry = OCEAN (Optimized Catheter Valvular Intervention)-Mitral Registry.

With the widespread adoption of M-TEER, iterative device improvements have culminated in the latest generation’s multiple clip size options and independent grasping mechanism. These advancements allow for tailored adaptation to the diverse anatomical variations of the mitral valve, enabling operators to select the optimal clip size and independently control leaflet grasping in complex cases, thereby achieving more reliable and effective MR reduction. The enhanced effectiveness of the newer-generation device has been corroborated by the EXPAND G4 studies,^5^^,^^6^ early findings from our registry,^7^ and other observational studies.^12^^,^^13^ This study uniquely provides a systematic comparison of baseline patient characteristics, anatomical details, procedural data, and long-term outcomes between the newer-generation and early-generation devices.

In line with previous studies,^5^^,^^7^ our findings showed that the newer-generation M-TEER device achieved shorter procedural and device times with fewer clips, resulting in a lower postprocedural TMPG, while providing effective MR reduction comparable to the early-generation device. The incidence of mechanical complications, including single leaflet device attachment, leaflet tear, and clip embolization, was notably low and further reduced with the newer-generation device. Moreover, effective MR reduction (MR ≤2+) was maintained in nearly 90% of DMR cases and 95% of FMR cases in both device generations at 1 year. A notable finding was that the newer-generation device was associated with a reduced risk of HF rehospitalization compared to the early-generation device, with clinical benefits more pronounced in patients with DMR than those with FMR.

Residual MR and elevated TMPG are known risk factors for future HF rehospitalization following M-TEER.^8^^,^^14^, ^15^, ^16^ Consistent with publised data, higher postprocedural MR and TMPG were linked to an increased risk of HF rehospitalization in this cohort. Whereas postprocedural MR grades did not differ significantly between device generations, the newer-generation device achieved a significantly lower postprocedural TMPG, likely due to the reduced number of clips. Intriguingly, our findings demonstrated that the lower HF rehospitalization risk with the newer-generation device was partially attributable to its ability to achieve a lower TMPG.

Other mechanisms underlying the reduced HF rehospitalization risk with the newer-generation device remain speculative. A key consideration is the lack of core-lab evaluation of MR severity in this study. Unlike baseline MR severity, the assessment of postprocedural MR severity after M-TEER primarily relies on qualitative methods due to challenges posed by the presence of the devices and multiple jet orifices.^17^ This qualitative approach introduces potential bias; even though the device advancements may have contributed to a more effective MR reduction, qualitative assessments—potentially influenced by relative comparisons with recent cases treated by the advanced technology—may inherently rate MR severity more stringently in the newer-generation devices, leading to an underestimation of the observed improvements. Indeed, the EXPAND G4 study, which used core lab echocardiographic assessment, suggested that the newer-generation device achieved more effective MR reduction than the early-generation device did.^5^ Consequently, the mediation effect of MR reduction on outcomes may not have been precisely estimated in our study.

Although the impact of postprocedural TMPG on HF rehospitalization risk showed no interaction with MR etiology—unlike findings in a previous study^14^—the impact of postprocedural MR severity on HF rehospitalization did vary by etiology. This suggests that if the MR reduction effect of the newer-generation device has indeed been underestimated, the greater protective effect seen in DMR patients may be attributable to a more effective MR reduction by the newer-generation device—though accurately assessing this effect was challenging in our study due to the absence of core laboratory, as discussed. This hypothesis warrants further investigation in studies using more rigorous MR severity assessments.

### Study limitations

The design of this before-and-after study may introduce bias due to temporal changes in clinical practice and procedural learning curve. During the 6-year study period, indications of M-TEER and optimal medical therapy regimens slightly shifted, as evidenced by differences in baseline characteristics. Although potential biases were carefully addressed using advanced statistical techniques, we cannot entirely rule out bias from unmeasured or unrecognized confounders, a limitation common to all observational studies. Additionally, as previously mentioned, echocardiographic data were systematically collected, but not adjudicated by a core laboratory, which necessitates caution when interpreting the findings. Further studies are warranted to corroborate these results and explore the mechanisms underlying the improved clinical outcomes associated with the newer-generation device.

## Conclusions

The newer-generation M-TEER system demonstrated reduced procedural time, fewer mechanical complications, and required a fewer clips, resulting in a lower TMPG while achieving effective MR reduction comparable to the early-generation system. Furthermore, the newer-generation device was associated with a reduced risk of HF rehospitalization with the protective effect more pronounced in patients with DMR compared to those with FMR.

## Funding Support and Author Disclosures

The OCEAN-Mitral registry, which is part of the OCEAN-SHD registry, is supported by Edwards Lifesciences, Medtronic Japan, Boston Scientific, Abbott Medical Japan, and Daiichi-Sankyo. Drs Saji, Kubo, Izumo, Watanabe, and Amaki have reported that they are clinical proctors of TEER for Abbott Medical; and have received consultant fees from Abbott Medical. Drs Asami and Kodama have reported that they are clinical proctors of TEER for Abbott Medical; and have received speaker fees from Abbott Medical. Dr Yamamoto has reported that he is a clinical proctor of TEER for Abbott Medical; and has received lecture fees from Abbott Medical. Dr Yamaguchi has reported that he is a clinical proctor of TEER for Abbott Medical; and has received lecture fees and a scholarship donation from Abbott Medical. Dr Ohno has reported that he is a clinical proctor of TEER for Abbott Medical; and has received consultant, advisor, and speaker fees from Abbott Medical. Drs Enta, Shirai, Mizuno, Ueno, Bota, and Hayashida have reported that they are clinical proctors of TEER for Abbott Medical. All other authors have reported that they have no relationships relevant to the contents of this paper to disclose.
