# Supplementary material for: Early- Versus Newer-Generation Transcatheter Mitral Valve Edge-to-Edge Repair Systems: Insights From the OCEAN-Mitral Registry
Source: JACC Asia. 2025 Jul 15;5(9):1110–20. doi: 10.1016/j.jacasi.2025.05.013 (PMC12426677; doi:10.1016/j.jacasi.2025.05.013)
Supplement: Supplemental Tables 1-5 and Supplemental Figures 1-3 [file mmc1.docx]

**Supplemental Online Content**

**Table of Contents**

[**Supplemental Table 1. Baseline variables included in the multivariable models in the DMR and FMR cohorts.** 2](#_Toc192105617)

[**Supplemental Table 2. Baseline characteristics in the DMR and FMR cohorts** 3](#_Toc192105618)

[**Supplemental Table 3. Optimal medical therapy in the FMR cohort** 4](#_Toc192105619)

[**Supplemental Table 4. Sensitivity analysis for clinical outcomes** 5](#_Toc192105620)

[**Supplemental Table 5. Standardized mean differences in pre- and post-matching cohorts** 6](#_Toc192105621)

[**Supplemental Figure 1. Subgroup analysis of the device-generation effect on clinical outcomes.** 7](#_Toc192105622)

[**Supplemental Figure 2. Mediation model between device generation and HF rehospitalization by TMPG and MR severity at discharge.** 8](#_Toc192105623)

[**Supplemental Figure 3. Subgroup analysis on the impact of post-procedural TMPG and MR on HF rehospitalization stratified by MR etiology.** 9](#_Toc192105624)

## **Supplemental Table 1. Baseline variables included in the multivariable models in the DMR and FMR cohorts.**

| **Multivariable model 1 (for DMR cohort)** |
| --- |
| age, sex, BMI, BSA, EuroSCORE II, Clinical frailty scale, NYHA III or IV, hypertension, diabetes, dyslipidemia, chronic kidney disease, atrial fibrillation, coronary artery disease, COPD, LVEF. |
| **Multivariable model 2 (for FMR cohort)** |
| age, sex, BMI, BSA, EuroSCORE II, Clinical frailty scale, NYHA III or IV, hypertension, diabetes, dyslipidemia, chronic kidney disease, atrial fibrillation, coronary artery disease, COPD, CIED, RASi, Beta blockers, MRA, SGLT2i, Loop diuretics, LVEF, LVEDV, LVESV, MR grade, Atrial MR. |
| DMR = degenerative mitral regurgitation; FMR = functional mitral regurgitation; BMI = body mass index; BSA = body surface area; NYHA = New York Heart Association; COPD = chronic obstructive pulmonary disease; CIED = cardiovascular implantable electronic device; RASi = renin-angiotensin system inhibitors; MRA = mineralocorticoid receptor antagonist; SGLT2i = sodium-glucose cotransporter-2 inhibitors; LVEF = left ventricular ejection fraction; LVEDV = left ventricular end-diastolic volume; LVESV = left ventricular end-systolic volume; MR = mitral regurgitation. |

## **Supplemental Table 2. Baseline characteristics in the DMR and FMR cohorts**

|  | **DMR cohort (N = 1,115)** | | | **FMR cohort (N = 2,623)** | | |
| --- | --- | --- | --- | --- | --- | --- |
|  | **Early-generation** | **Newer-generation** | **p-value** | **Early-**  **generation** | **Newer-generation** | **p-value** |
|  | N = 447 | N = 668 |  | N = 1,034 | N = 1,589 |  |
|  |  |  |  |  |  |  |
| Age (years) | 80 [73-85] | 81 [74-86] | 0.010 | 78 [71-84] | 79 [72-84] | 0.007 |
| Sex (male) | 212 (47.4%) | 287 (43.0%) | 0.158 | 621 (60.1%) | 935 (58.8%) | 0.542 |
| Body mass index (kg/cm²) | 20.6 [18.6-23.3] | 20.8 [18.5-22.9] | 0.594 | 21.2 [18.9-23.5] | 21.1 [18.8-23.4] | 0.546 |
| Body surface area (m2) | 1.45 [1.32-1.59] | 1.44 [1.31-1.56] | 0.547 | 1.53 [1.39-1.65] | 1.53 [1.38-1.67] | 0.680 |
| EuroSCORE II | 4.09 [2.59-6.50] | 4.02 [2.66-6.27] | 0.873 | 5.29 [3.34-8.72] | 5.97 [3.74-10.56] | <0.001 |
| Clinical Frailty Scale | 4 [3-5] | 4 [3-5] | 0.026 | 4 [3-4] | 4 [3-4] | 0.817 |
| NYHA III or IV | 280 (62.6%) | 383 (57.3%) | 0.082 | 677 (65.5%) | 1,022 (64.3%) | 0.558 |
| **Concomitant diseases** |  |  |  |  |  |  |
| Hypertension | 311 (69.6%) | 463 (69.3%) | 0.947 | 688 (66.5%) | 972 (61.2%) | 0.005 |
| Diabetes mellitus | 66 (14.8%) | 88 (13.2%) | 0.479 | 351 (33.9%) | 478 (30.1%) | 0.039 |
| Dyslipidemia | 171 (38.3%) | 252 (37.7%) | 0.900 | 582 (56.3%) | 804 (50.6%) | 0.004 |
| Chronic kidney disease (GFR<60) | 376 (84.1%) | 558 (83.5%) | 0.868 | 903 (87.3%) | 1,401(88.2%) | 0.541 |
| Atrial fibrillation | 274 (61.3%) | 365 (54.6%) | 0.031 | 669 (64.7%) | 995 (62.6%) | 0.281 |
| Coronary artery disease | 97 (21.7%) | 119 (17.8%) | 0.122 | 449 (43.4%) | 641 (40.3%) | 0.123 |
| COPD | 43 (9.6%) | 57 (8.5%) | 0.593 | 110 (10.6%) | 111 (7.0%) | 0.001 |
| DMR = degenerative mitral regurgitation; FMR = functional mitral regurgitation; NYHA = New York Heart Association; eGFR = estimated glomerular filtration rate; COPD = chronic obstructive pulmonary disease. | | | | | | |

## **Supplemental Table 3. Optimal medical therapy in the FMR cohort**

|  | **FMR cohort (N = 2,623)** | | |
| --- | --- | --- | --- |
|  | **Early-generation** | **Newer-generation** | **p-value** |
|  | N = 1,034 | N = 1,589 |  |
|  |  |  |  |
| **Cardiovascular Implantable Electronic Device** |  |  | 0.150 |
| Pacemaker | 71 (6.9%) | 136 (8.6%) |  |
| ICD | 67 (6.5%) | 95 (6.0%) |  |
| CRT-P/D | 156 (15.0%) | 196 (12.3%) | 0.046 |
| **Medication** |  |  |  |
| RASI | 670 (65.0%) | 1031 (65.5%) | 0.833 |
| ACEI | 424 (41.2%) | 407 (25.9%) | <0.001 |
| ARB | 248 (24.0%) | 303 (19.1%) | <0.001 |
| ARNI | 1 (0.1%) | 328 (20.7%) | <0.001 |
| MRA | 588 (57.0%) | 949 (59.9%) | 0.133 |
| Beta-blocker | 850 (83.3%) | 1246 (79.3%) | 0.011 |
| SGLT2 inhibitor | 104 (10.1%) | 563 (35.4%) | <0.001 |
| Loop diuretics | 894 (86.5%) | 1,243 (78.2%) | <0.001 |
| FMR = functional mitral regurgitation; ICD implantable cardioverter defibrillator; CRT-P/D = cardiac resynchronization therapy – pacemaker/defibrillator; RASI = renin-angiotensin system inhibitors; ACEI = angiotensin-converting enzyme inhibitors; ARB = angiotensin II receptor blocker; ARNI = angiotensin receptor-neprilysin inhibitors; MRA = mineralocorticoid receptor antagonist; SGLT2 = sodium-glucose cotransporter-2. | | | |

## **Supplemental Table 4. Sensitivity analysis for clinical outcomes**

|  | **Excluding cases in 2018** | | **Propensity score-matched*** | |
| --- | --- | --- | --- | --- |
|  | **HR (95% CI)** | **p-value** | **HR (95% CI)** | **p-value** |
| **DMR cohort** | N=1,011 |  | N=752 |  |
| Composite endpoint of mortality and HF rehospitalization | 0.68 (0.52-0.90) | 0.006 | 0.66 (0.49-0.89) | 0.006 |
| All-cause mortality | 0.74 (0.53-1.02) | 0.067 | 0.77 (0.54-1.09) | 0.142 |
| Cardiovascular mortality | 0.75 (0.48-1.17) | 0.203 | 0.74 (0.45-1.23) | 0.247 |
| HF rehospitalization | 0.52 (0.35-0.79) | 0.002 | 0.47 (0.30-0.75) | 0.001 |
| **FMR cohort** | N=2,382 |  | N=1,344 |  |
| Composite endpoint of mortality and HF rehospitalization | 0.99 (0.86-1.14) | 0.884 | 0.93 (0.78-1.11) | 0.405 |
| All-cause mortality | 1.22 (1.02-1.46) | 0.032 | 1.11 (0.89-1.39) | 0.337 |
| Cardiovascular mortality | 1.24 (0.98-1.56) | 0.073 | 0.97 (0.73-1.28) | 0.812 |
| HF rehospitalization | 0.75 (0.62-0.90) | 0.002 | 0.74 (0.59-0.93) | 0.001 |
|  |  |  |  |  |
| HR = hazard ratio; CI = confidence interval; DMR = degenerative mitral regurgitation; FMR = functional mitral regurgitation; HF = heart failure. * The propensity score was estimated using a multivariable logistic regression model, incorporating the same variables used in the Cox multivariable adjustment models (see Supplemental Table 1). Matching was performed using a 1:1 greedy nearest-neighbor algorithm, with a caliper width of 0.2. After matching, the early-generation group and the newer-generation group were well balanced, with an ASD <0.10 across nearly all measured baseline characteristics, except for EuroSCORE II in the DMR cohort (SMD = 0.137) (see Supplemental Table 5). | | | | |

## **Supplemental Table 5. Standardized mean differences in pre- and post-matching cohorts**

|  | **DMR cohort** | | **FMR cohort** | |
| --- | --- | --- | --- | --- |
|  | **Pre-matching** | **Post-matching** | **Pre-matching** | **Post-matching** |
| Number | 447 vs. 668 | 376 vs. 376 | 1,034 vs. 1589 | 672 vs. 672 |
|  |  |  |  |  |
| Age (years) | 0.083 | 0.025 | 0.071 | 0.032 |
| Sex (male) | 0.090 | 0.011 | 0.025 | 0.003 |
| Body mass index (kg/cm²) | 0.027 | 0.067 | 0.028 | 0.046 |
| Body surface area (m2) | 0.045 | 0.042 | 0.019 | 0.026 |
| EuroSCORE II | 0.063 | 0.137 | 0.137 | 0.011 |
| Clinical Frailty Scale | 0.121 | 0.074 | 0.006 | 0.026 |
| NYHA III or IV | 0.108 | 0.006 | 0.024 | 0.028 |
| **Concomitant diseases** |  |  |  |  |
| Hypertension | 0.006 | 0.035 | 0.112 | 0.031 |
| Diabetes mellitus | 0.055 | 0.030 | 0.124 | 0.088 |
| Dyslipidemia | 0.011 | 0.005 | 0.114 | 0.006 |
| Chronic kidney disease (GFR<60) | 0.016 | 0.030 | 0.026 | 0.037 |
| Atrial fibrillation | 0.135 | 0.033 | 0.043 | 0.006 |
| Coronary artery disease | 0.098 | 0.020 | 0.063 | 0.006 |
| COPD | 0.038 | 0.018 | 0.129 | <0.001 |
| Cardiovascular Implantable Electronic Device | NA | NA | 0.104 | 0.061 |
| **Medication** |  |  |  |  |
| RASI | NA | NA | 0.010 | 0.031 |
| MRA | NA | NA | 0.060 | 0.003 |
| Beta-blocker | NA | NA | 0.105 | 0.024 |
| SGLT2 inhibitor | NA | NA | 0.635 | 0.023 |
| Loop diuretics | NA | NA | 0.217 | 0.021 |
| **Echocardiography** |  |  |  |  |
| MR grade | NA | NA | 0.163 | 0.016 |
| Atrial FMR | NA | NA | 0.073 | 0.008 |
| LVEF (%) | NA | NA | 0.009 | 0.012 |
| LVESV, mL | NA | NA | 0.036 | 0.012 |
| LVEDV, mL | NA | NA | 0.066 | 0.015 |
| DMR = degenerative mitral regurgitation; FMR = functional mitral regurgitation; NYHA = New York Heart Association; eGFR = estimated glomerular filtration rate; COPD = chronic obstructive pulmonary disease. RASI = renin-angiotensin system inhibitors; MRA = mineralocorticoid receptor antagonist; SGLT2 = sodium-glucose cotransporter-2; LVEF = left ventricular ejection fraction; LVESV = left ventricular end-systolic volume; LVEDV = left ventricular end-diastolic volume. | | | | |

## **Supplemental Figure 1. Subgroup analysis of the device-generation effect on clinical outcomes.**

**
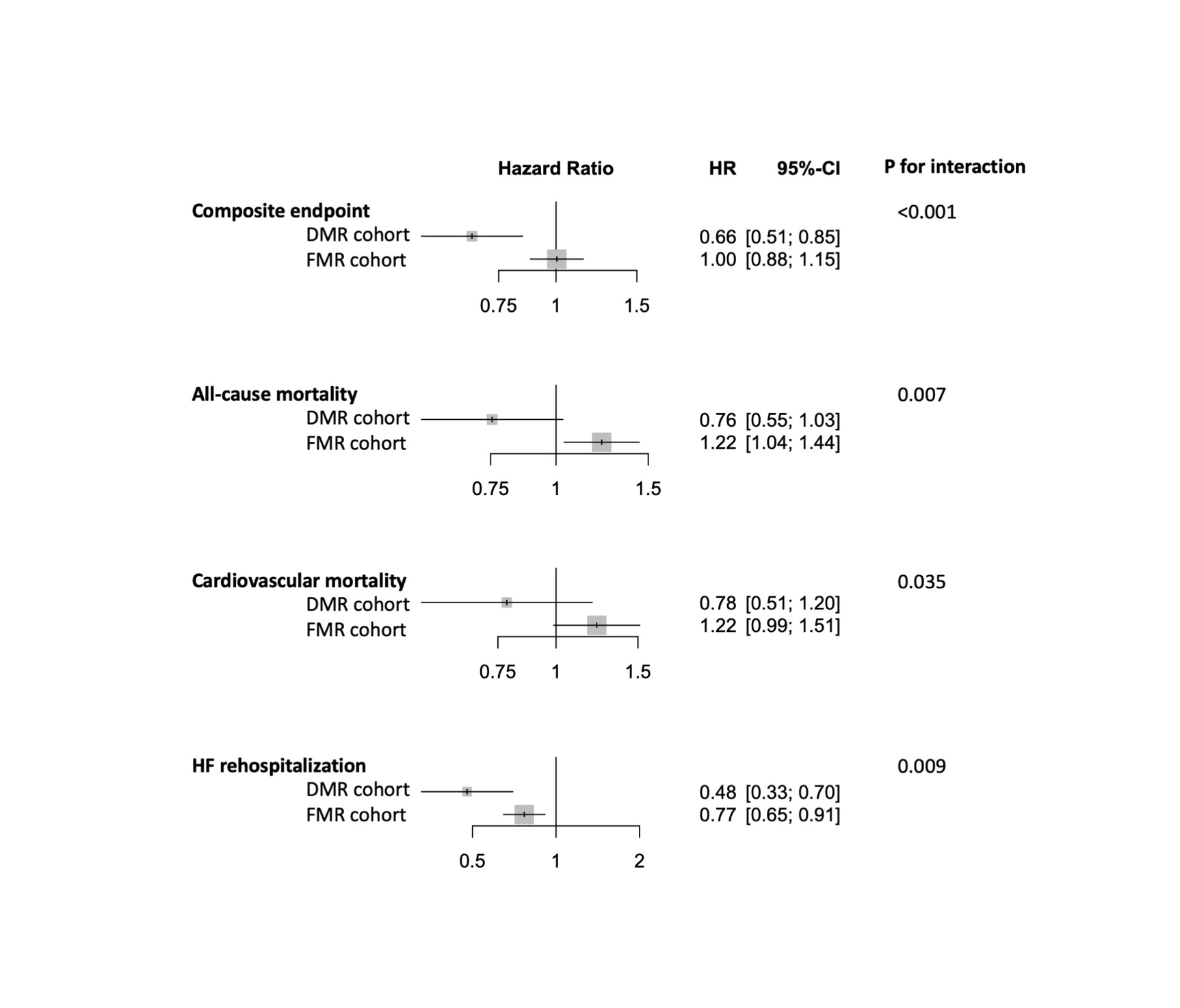
**Significant interactions were observed between the etiology of mitral regurgitation and the effect of device generation across all clinical endpoints.
HR = hazard ratio; DMR = degenerative mitral regurgitation; FMR = functional mitral regurgitation; HF = heart failure.

## **Supplemental Figure 2. Mediation model between device generation and HF rehospitalization by TMPG and MR severity at discharge.**


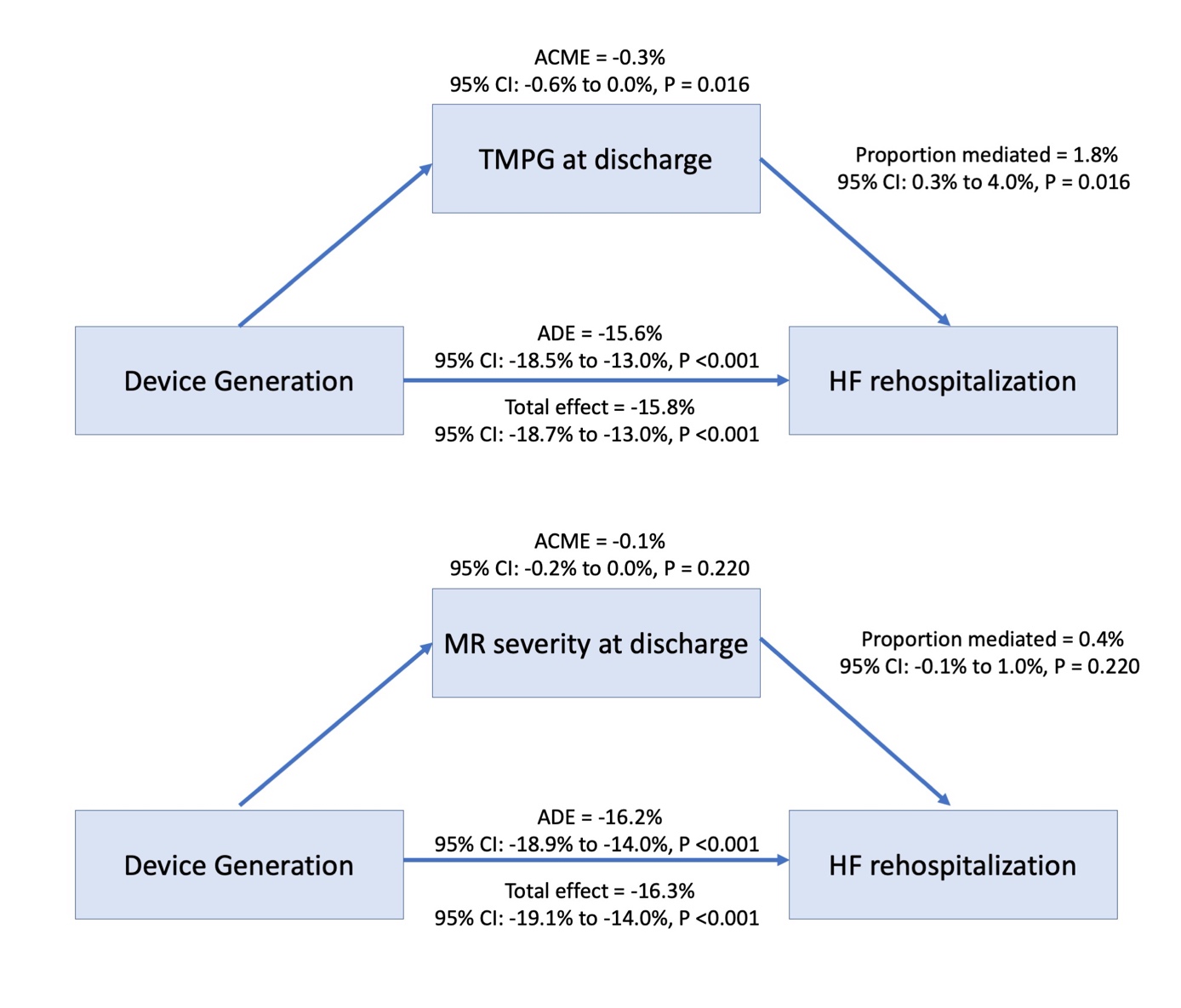
 The association between device generation and HF rehospitalization is significantly mediated by post-procedural TMPG but not by post-procedural MR severity. Outliers in post-procedural TMG were identified and excluded using the Smirnov-Grubbs test.
HF = heart failure; TMPG = trans-mitral mean pressure gradient; MR = mitral regurgitation; ACME = average causal mediation effect; ADE = average direct effect.

## **Supplemental Figure 3. Subgroup analysis on the impact of post-procedural TMPG and MR on HF rehospitalization stratified by MR etiology.**


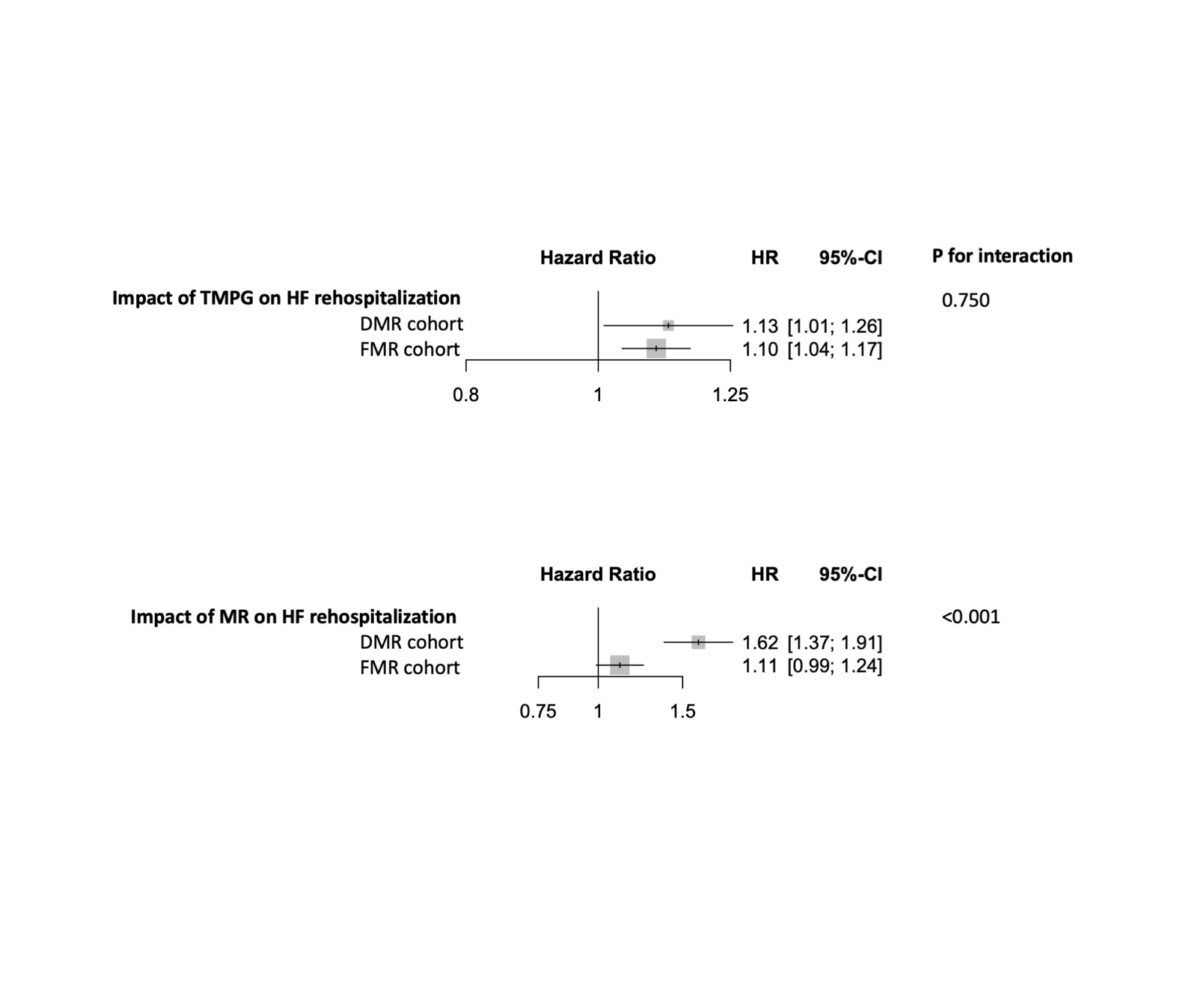

Significant interaction was observed between the etiology of MR and the effect of post-procedural MR severity, but not with post-procedural TMPG. Outliers in post-procedural TMG were identified and excluded using the Smirnov-Grubbs test.
HR = hazard ratio; TMPG = trans-mitral mean pressure gradient; MR = mitral regurgitation; DMR = degenerative mitral regurgitation; FMR = functional mitral regurgitation; HF = heart failure.
